# Supplementary material for: Quantifying defective and wild-type viruses from high-throughput RNA sequencing
Source: Bioinformatics. 2024 Nov 4;40(11):btae651. doi: 10.1093/bioinformatics/btae651 (PMC11583936; doi:10.1093/bioinformatics/btae651)
Supplement: btae651_Supplementary_Data [file btae651_supplementary_data.pdf]

Quantifying defective and wild-type viruses from high-throughput RNA sequencing

Juan C. Muñoz-Sánchez†, María J. Olmo-Uceda, José-Ángel Oteo and Santiago F. Elena

† Correspondence: jc.munoz@csic.es

Appendices

A. SDgenerator

SDgenerator v.2 is a Python program developed to simulate short-read RNA-seq samples formed by controlled populations of wt virus and its associated DVGs. The algorithm takes as input the reference genome (*i.e.*, the wt virus) and a table with the DVG composition with the next information: DVG type, BP, RI, and proportion of each DVG in the sample (p_i^{nom}). The user can control for the library size (N_T, total reads) and the length of the reads (L) of the synthetic sample. The code is available at `https://github.com/MJmaolu/SDgenerator/tree/main`.

A

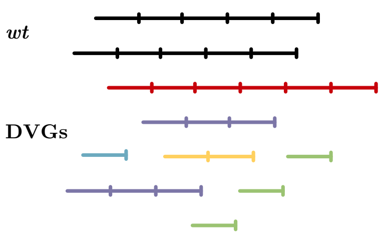

B **C** **D**

DVG type	BP	RI	$p_i^{nom}(\%)$	l_i	N_i
wt	-	-	20	5L	10
**dvg 1**	bp_1	ri_1	10	6L	6
**dvg 2**	bp_2	ri_2	20	3L	6
**dvg 3**	bp_3	ri_3	10	1L	1
**dvg 4**	bp_4	ri_4	10	2L	2
**dvg 5**	bp_5	ri_5	30	1L	3

E

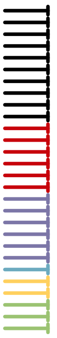

Fig. A1: Schematic pipeline of SDgenerator. (A) Expected genome composition. For simplicity a population of only six different genomes is represented. Black genomes are the wt and colored genomes represent different DVGs. The segments indicate arbitrary unites of length. (B) Genomes in (A) represented as input table for SDgenerator, the number of genomes of each type are indicated as proportion in the total sample (p_i^{nom}). (C) Reconstructed length of each genome (l_i). (D) Finally, the number of reads to generate a sample with $N_T = 28$ and $L = 1$ is calculated (A1) and (E) the program launches the generation of synthetic reads with wgsim and concatenates the results in a single fastq file.

Being N_T the desired total number of reads with length L, N_T the total number of genomes, l_i the length of each genome with proportion p_i^{nom}, the total number of reads associated with each genome N_i is:

$$N_i = N_T \frac{p_i^{nom} l_i}{\sum_i p_i^{nom} l_i}. \tag{A1}$$

Proof:
User inputs: N_T and p_i^{nom} (from which l_i is derived knowing BP and RI). The number of reads to assign to each genome g_i (N_i) is the amount of copies of that genome (n_i) times its length (l_i) divided by the length of the read (L):

$$N_i = n_i \frac{l_i}{L}. \tag{A2}$$

The number of genomes of each specie (n_i) can be obtained knowing the total number of genomes and the proportion of the sample corresponding a g_i:

$$n_i = p_i^{nom} n_T. \tag{A3}$$

The total number of genomes in the sample can be obtained starting from the total number of reads (N_T) and expressing it as the sum of the reads assigned to each individual genome. If this individual contribution is written in terms of the proportion of each genome in the sample and the total number of samples then n_T can be isolated as follows:

$$N_T = \sum_i N_i = \frac{1}{L}\sum_i n_i l_i = \frac{n_T}{L}\sum_i p_i^{nom} l_i \Leftrightarrow \frac{n_T}{L} = \frac{N_T}{\sum_i p_i^{nom} l_i}. \tag{A4}$$

From (A2) - (A4) one can easily obtain (A1).

Once each genome has been reconstructed (an saved in fasta format) and its N_i has been calculated, SDgeneratoruses the tool wgsim (`https://github.com/lh3/wgsim`) to simulate the sequence reads and concatenate them in a single fastq file. We set by default all the mutation and error rates to 0 but both can be modified in the source code.

B. Contribution matrix for Sec. 3.3

$$\vec{b} = (1 \ldots 2000\ 2001 \ldots 2200\ 2201 \ldots 2999\ 3000 \ldots 3100\ 3101 \ldots 3499\ 3500 \ldots 4099\ 4100 \ldots 4900\ 4901 \ldots 5500\ 5501 \ldots 7000\ 7001 \ldots 7999\ 8000 \ldots 8749\ 8750 \ldots 8999\ 9000 \ldots 9832) \quad \text{(B1)}$$

$$\mathcal{C} = \begin{pmatrix}
1 & \ldots & 1\,1 & \ldots & 1\,1 & \ldots & 1\,1 & \ldots & 1\,1 & \ldots & 1\,1 & \ldots & 1\,1 & \ldots & 1\,1 & \ldots & 1\,1 & \ldots & 1\,1 & \ldots & 1\,1 & \ldots & 1\,1 & \ldots & 1\,1 & \ldots & 1 \\
1 & \ldots & 1\,0 & \ldots & 0\,0 & \ldots & 0\,1 & \ldots & 1\,1 & \ldots & 1\,1 & \ldots & 1\,1 & \ldots & 1\,1 & \ldots & 1\,1 & \ldots & 1\,1 & \ldots & 1\,1 & \ldots & 1\,1 & \ldots & 1\,1 & \ldots & 1 \\
1 & \ldots & 1\,1 & \ldots & 1\,1 & \ldots & 1\,1 & \ldots & 1\,1 & \ldots & 1\,1 & \ldots & 1\,1 & \ldots & 1\,1 & \ldots & 1\,1 & \ldots & 1\,0 & \ldots & 0\,0 & \ldots & 0\,0 & \ldots & 0\,1 & \ldots & 1 \\
0 & \ldots & 0\,0 & \ldots & 0\,0 & \ldots & 0\,0 & \ldots & 0\,0 & \ldots & 0\,0 & \ldots & 0\,0 & \ldots & 0\,0 & \ldots & 0\,0 & \ldots & 0\,0 & \ldots & 0\,1 & \ldots & 1\,2 & \ldots & 2\,2 & \ldots & 2 \\
2 & \ldots & 2\,2 & \ldots & 2\,1 & \ldots & 1\,1 & \ldots & 1\,0 & \ldots & 0\,0 & \ldots & 0\,0 & \ldots & 0\,0 & \ldots & 0\,0 & \ldots & 0\,0 & \ldots & 0\,0 & \ldots & 0\,0 & \ldots & 0\,0 & \ldots & 0 \\
1 & \ldots & 1\,1 & \ldots & 1\,1 & \ldots & 1\,1 & \ldots & 1\,1 & \ldots & 1\,2 & \ldots & 2\,2 & \ldots & 2\,2 & \ldots & 2\,1 & \ldots & 1\,1 & \ldots & 1\,1 & \ldots & 1\,1 & \ldots & 1\,1 & \ldots & 1 \\
1 & \ldots & 1\,1 & \ldots & 1\,1 & \ldots & 1\,1 & \ldots & 1\,1 & \ldots & 1\,1 & \ldots & 1\,2 & \ldots & 2\,1 & \ldots & 1\,1 & \ldots & 1\,1 & \ldots & 1\,1 & \ldots & 1\,1 & \ldots & 1\,1 & \ldots & 1
\end{pmatrix} \quad \text{(B2)}$$

C. Pre-processing algorithm. DVGfinder output table curation.

DVGfinder performs an intensive search on reads that do not match with the reference genome and classifies them into the most suitable category. This intensive search outcomes with a large table of candidate DVGs which needs to be curated. Here we present the pipeline we used to keep only those DVGs which events appear represented into the depth profile as a measurable change in the median of the plateaus adjacent to that DVG event. The pre-processing steps are the following:

(i) Filtering
Only those DVG candidates which number of reads conted by ViReMa-a outcomes a threshold, R_{th}, are kept. Here we set this threshold relative to the maximum depth of the profile, $R_{th} = 0.005 \cdot \max(\vec{d})$. An example of the DVGfinder output table after this filtering step can be found at Table D1.

(ii) Merging rows
Rows which share the same DVG type and their positions differ below a certain threshold, Δ_b, are merged. (here, $\Delta_b = 10$). The resulting position will be the weighted mean (according to the number of ViReMa-a reads) of all merged rows. The associated number of reads will be the sum of the merged ones.

(iii) Select event position with relevant change within plateaus' medians
Let's call $\vec{X}$ to the event position vector in which all DVG events (BP and RI) positions are stored. To avoid short plateau's, positions in $\vec{X}$ differing less than $\Delta_b/2$ are combined keeping the mean value. $\vec{X}$ defines the limits of the plateaus. In this step we will remove all event positions which adjacent plateau's median do not differ more than a certain threshold relative to the noise level.

(a) Obtain the noise level of the depth profile. The noise level computation follows the same procedure as in Sec. **??**. Here, the noise level is computed as $n_l = (|u_b| + |l_b|)/2$, being u_b and l_b the upper and lower bounds of the relative depth deviation to the median.

(b) Compute the relative difference in consecutive plateau's median values, $\Delta\tilde{d}_{i,j} = |\tilde{d}_i - \tilde{d}_j|/\tilde{d}_i$.

(c) Keep those positions which plateau's median depth at one side of the splitter and the other is greater than certain threshold relative to the noise level, $\Delta_{\tilde{d}}$. This is, keep positions in $\vec{X}(i) \iff \min(\Delta\tilde{d}_{i,i+1}, \Delta\tilde{d}_{i-1,i}) > \Delta_{\tilde{d}} \cdot n_l$. Here, $\Delta_{\tilde{d}} = 0.5$.

(iv) Filter DVGs without their BP or RI position present at the curated $\vec{X}$
After having joined plateaus, it is possible that some DVGs do not have its start or ending position in $\vec{X}$. In this final step we will discard those DVGs as they not contribute to the depth profile. In this step, a threshold on the minimum event size, |BP−RI|, can be set.

The pre-procesing result reduces the number of DVGs of the sample from 8500 from the DVGfinder raw output to 34. The main reduction occurs during the filtering step. Fig. C1 show the depth profile with the event positions remaining after the pre-processing. Depending on the user's motivation for the analysis, a different choice of parameters may be more appropriate. In case the goal is to have more power detecting DVGs, we will relax the parameters R_{th} and $\Delta_{\tilde{d}}$. Finding the most restrictive parameters that still allow us to reconstruct this depth profile would be the most conservative option and most likely to give us DVGs actually present in the sample.

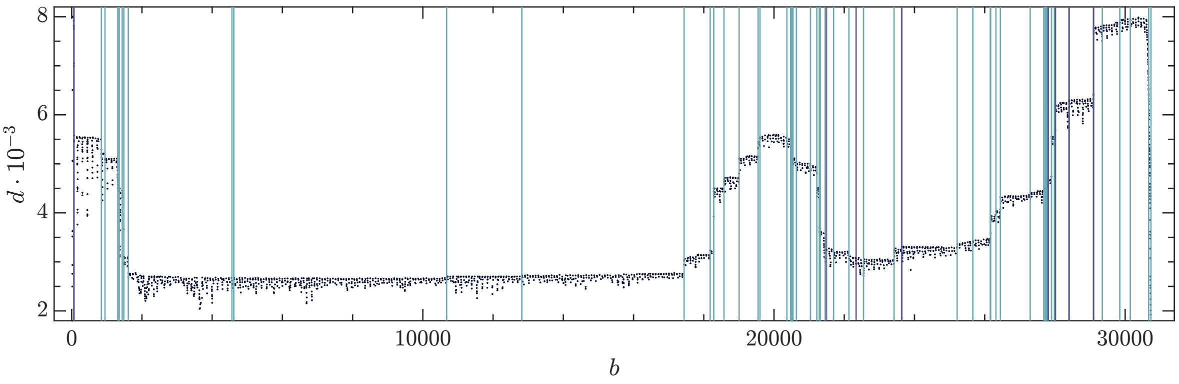

Fig. C1: Depth profile with DVGs event's positions indicated as blue lines. Purple lines indicate the localization of the known subgenomic positions for HCoV-OC43.

D. Case study: input data and results.

Table D1. DVGfinder output tables using `metasearch` mode. DVG which appear less than R_{th} times, with $R_{th} = 0.005 \cdot \max(\vec{d})$, have been filtered.

Type	Start	End
deletion	1311	19006
deletion	1312	19007
deletion	1352	18574
deletion	1353	18575
deletion	1437	18181
deletion	1438	18182
deletion	1483	18282
deletion	1484	18283
deletion	1611	17438
deletion	1613	17440
deletion	20184	27338
deletion	20186	27340
deletion	20266	27986
deletion	20367	26158
deletion	20368	26159
deletion	20474	27740
deletion	20486	23419
deletion	20493	27296
deletion	20524	25661
deletion	20541	27682
deletion	20637	26319
deletion	20638	26320
deletion	21037	27899

Type	Start	End
deletion	21218	26168
deletion	21220	26170
deletion	21290	27905
deletion	21315	25214
deletion	21317	25216
deletion	21457	26445
deletion	21458	26446
deletion	21696	29347
deletion	22134	27814
deletion	23530	23532
deletion	27749	27770
deletion	27751	27772
deletion	27983	27996
deletion	4563	4615
deletion	57	28009
deletion	58	29097
deletion	59	28010
deletion	60	12818
deletion	61	18672
deletion	61	28013
deletion	61	28402
deletion	62	10677
deletion	66	23640

Type	Start	End
deletion	21039	27901
deletion	68	27724
deletion	70	21500
deletion	790	19693
deletion	811	813
deletion	813	19197
deletion	814	19198
deletion	832	17522
deletion	844	19545
deletion	848	19549
deletion	948	19600
deletion	20816	20818
deletion	23527	23529
insertion	1082	1082
insertion	12157	12157
insertion	3	22549
insertion	22648	22648
insertion	29230	29351
insertion	29847	30146
insertion	30669	30735
insertion	12152	12152

Table D2. Result of the case study. n_i has been rounded to the nearest integer. Subgenomics appear colored in the table.

Type	Start	End	$p_i(\%)$	Reads	n_i
deletion	1312	19007	5.36	2916	421
deletion	1353	18575	2.61	1071	205
deletion	1438	18182	0.95	220	75
deletion	1484	18283	16.56	3066	1301
deletion	1612	17439	5.22	582	410
deletion	20368	26159	0.96	140	76
deletion	20474	27740	0.16	139	13
deletion	20486	23419	2.87	920	225
deletion	20493	27296	1.07	52	84
deletion	20524	25661	0.60	100	45
deletion	20541	27682	0.49	49	39
deletion	20638	26320	1.12	900	88
deletion	21038	27903	0.75	156	59
deletion	21219	26169	5.35	2852	421
deletion	21290	27903	10.50	3603	825
deletion	21316	25215	1.29	360	101
deletion	21458	26446	3.99	789	313

Type	Start	End	$p_i(\%)$	Reads	n_i
deletion	21696	29349	0.98	46	77
deletion	22134	27814	1.55	257	122
deletion	27750	27771	0.13	402	10
deletion	27985	27996	0.25	772	20
deletion	4563	4615	0.34	81	26
deletion	63	28010	8.42	1309	661
deletion	63	29098	18.91	5437	1486
deletion	63	12818	0.35	58	28
deletion	63	28402	1.23	201	97
deletion	63	10677	0.36	158	29
deletion	63	23640	0.25	71	20
deletion	63	27724	0.81	82	64
deletion	846	19547	3.96	1716	311
deletion	948	19600	1.57	652	123
insertion	1	22549	0.45	177	35
insertion	29847	30146	0.56	44	44
insertion	30669	30735	10^{-22}	55	0
